# Generalized lessons about sequence learning from the study of the serial reaction time task

**DOI:** 10.2478/v10053-008-0113-1

**Published:** 2012-05-21

**Authors:** Hillary Schwarb, Eric H. Schumacher

**Affiliations:** School of Psychology, Georgia Institute of Technology, Atlanta, Georgia, USA

**Keywords:** sequence learning, implicit learning, serial reaction time task

## Abstract

Over the last 20 years researchers have used the serial reaction time (SRT) task to investigate the nature of spatial sequence learning. They have used the task to identify the locus of spatial sequence learning, identify situations that enhance and those that impair learning, and identify the important cognitive processes that facilitate this type of learning. Although controversies remain, the SRT task has been integral in enhancing our understanding of implicit sequence learning. It is important, however, to ask what, if anything, the discoveries made using the SRT task tell us about implicit learning more generally. This review analyzes the state of the current spatial SRT sequence learning literature highlighting the stimulus-response rule hypothesis of sequence learning which we believe provides a unifying account of discrepant SRT data. It also challenges researchers to use the vast body of knowledge acquired with the SRT task to understand other implicit learning literatures too often ignored in the context of this particular task. This broad perspective will make it possible to identify congruences among data acquired using various different tasks that will allow us to generalize about the nature of implicit learning.

## Introduction

Learning is an integral part of human experience. Throughout our lives we are constantly presented with new information that mustbe attended, integrated, and stored. When learning is successful, the knowledge we acquire can be applied in future situations to improve and enhance our behaviors. Learning can occur both consciously and outside of our awareness. This learning without awareness, or *implicit learning*, has been a topic of interest and investigation for over 40 years (e.g., Thorndike & Rock, 1934). Many paradigms have been used to investigate implicit learning (cf. Cleeremans, Destrebecqz, & Boyer, 1998; Clegg, DiGirolamo, & Keele, 1998; Dienes & Berry, 1997), and one of the most popular and rigorously applied procedures is the serial reaction time (SRT) task. The SRT task is designed specifically to address issues related to learning of sequenced information which is central to many human behaviors (Lashley, 1951) and is the focus of this review (cf. also Abrahamse, Jiménez, Verwey, & Clegg, 2010).

Since its inception, the SRT task has been used to understand the underlying cognitive mechanisms involved in implicit sequence learning. In our view, the last 20 years can be organized into two main thrusts of SRT research: (a) research that seeks to identify the underlying locus of sequence learning; and (b) research that seeks to identify the role of divided attention on sequence learning in multi-task situations. Both pursuits teach us about the organization of human cognition as it relates to learning sequenced information and we believe that both also lead to the same conclusion. Namely, that sequence learning, both alone and in multi-task situations, largely involves stimulus-response associations and relies on response-selection processes. In this review we seek (a) to introduce the SRT task and identify important considerations when applying the task to specific experimental goals, (b) to outline the prominent theories of sequence learning both as they relate to identifying the underlying locus of learning and to understand when sequence learning is likely to be successful and when it will likely fail, and finally (c) to challenge researchers to take what has been learned from the SRT task and apply it to other domains of implicit learning to better understand the generalizability of what this task has taught us.

## The Serial Reaction Time Task

In 1987, Nissen and Bullemer developed a procedure for studying implicit learning that over the next two decades would become a paradigmatic task for studying and understanding the underlying mechanisms of spatial sequence learning: the SRT task. The goal of this seminal study was to explore learning without awareness. In a series of experiments, Nissen and Bullemer used the SRT task to understand the differences between single- and dual-task sequence learning. Experiment 1tested the efficacy of their design. On each trial, an asterisk appeared at one of four possible target locations each mapped to a separate response button (compatible mapping). Once a response was made the asterisk disappeared and 500 ms later the next trial began. There were two groups of subjects. In the first group, the presentation order of targets was random with the constraint that an asterisk could not appear in the same location on two consecutive trials. In the second group, the presentation order of targets followed a sequence composed of 10 target locations that repeated 10 times over the course of a block (i.e., “4-2-3-1-3-2-4-3-2-1” with *1*, *2*, *3*, and *4* representing the four possible target locations). Participants performed this task for eight blocks. Significant Block × Group interactions were observed in both the reaction time (RT) and accuracy data with participants in the sequenced group responding more quickly and more accurately than participants in the random group. This is the standard sequence learning effect. Participants who are exposed to an underlying sequence perform more quickly and more accurately on sequenced trials compared to random trials presumably because they are able to use knowledge of the sequence to perform more efficiently. When asked, 11 of the 12 participants reported having noticed a sequence, thus indicating that learning did not occur outside of awareness in this study. However, in Experiment 4 individuals with Korsakoff’s syndrome performed the SRT task and did not notice the presence of the sequence. Data indicated successful sequence learning even in these amnesic patents. Thus, Nissen and Bullemer concluded that implicit sequence learning can indeed occur under single-task conditions.

In Experiment 2, Nissen and Bullemer (1987) again asked participants to perform the SRT task, but this time their attention was divided by the presence of a secondary task. There were three groups of participants in this experiment. The first performed the SRT task alone as in Experiment 1 (single-task group). The other two groups performed the SRT task and a secondary tone-counting task concurrently. In this tone-counting task either a high or low pitch tone was presented with the asterisk on each trial. Participants were asked to both respond to the asterisk location and to count the number of low pitch tones that occurred over the course of the block. At the end of each block, participants reported this number. For one of the dual-task groups the asterisks again followed a 10-position sequence (dual-task sequenced group) while the other group saw randomly presented targets (dual-task random group). There were a total of four blocks of 100 trials each. A significant Block × Group interaction resulted from the RT data indicating that the single-task group was faster than both of the dual-task groups. Post hoc comparisons revealed no significant difference between the dual-task sequenced and dual-task random groups. Thus these data suggested that sequence learning does not occur when participants cannot fully attend to the SRT task.

Nissen and Bullemer’s (1987) influential study demonstrated that implicit sequence learning can indeed occur, but that it may be hampered by multi-tasking. These studies spawned decades of research on implicit sequence learning using the SRT task investigating the role of divided attention in successful learning. These studies sought to explain both what is learned during the SRT task and when specifically this learning can occur. Before we consider these issues further, however, we feel it is important to more fully explore the SRT task and identify those considerations, modifications, and improvements that have been made since the task’s introduction.

## Methodological Considerations in the SRT task

Research has suggested that implicit and explicit learning rely on different cognitive mechanisms (N. J. Cohen & Eichenbaum, 1993; A. S. Reber, Allen, & Reber, 1999) and that these processes are distinct and mediated by different cortical processing systems (Clegg et al., 1998; Keele, Ivry, Mayr, Hazeltine, & Heuer, 2003; A. S. Reber et al., 1999). Therefore, a primary concern for many researchers using the SRT task is to optimize the task to extinguish or minimize the contributions of explicit learning. One aspect that seems to play an important role is the choice of sequence type.

### Sequence structure

In their original experiment, Nissen and Bullemer (1987) used a 10-position sequence in which some positions consistently predicted the target location on the next trial, whereas other positions were more ambiguous and could be followed by more than one target location. This type of sequence has since become known as a *hybrid sequence* (A. Cohen, Ivry, & Keele, 1990). After failing to replicate the original Nissen and Bullemer experiment, A. Cohen et al. (1990; Experiment 1)began to investigate whether the structure of the sequence used in SRT experiments affected sequence learning. They examined the influence of various sequence types (i.e., unique, hybrid, and ambiguous) on sequence learning using a dual-task SRT procedure. Their unique sequence included five target locations each presented once during the sequence (e.g., “1-4-3-5-2”; where the numbers *1*-*5* represent the five possible target locations). Their ambiguous sequence was composed of three possible target locations each of which was repeated exactly twice in the sequence (e.g., “2-1-3-2-3-1”). Finally, their hybrid sequence included four possible target locations and the sequence was six positions long with two positions repeating once and two positions repeating twice (e.g., “1-2-3-2-4-3”). They demonstrated that participants were able to learn all three sequence types when the SRT task was performed alone, however, only the unique and hybrid sequences were learned in the presence of a secondary tone-counting task. They concluded that ambiguous sequences cannot be learned when attention is divided because ambiguous sequences are complex and require attentionally demanding hierarchic coding to learn. Conversely, unique and hybrid sequences can be learned via simple associative mechanisms that require minimal attention and therefore can be learned even with distraction.

The effect of sequence structure was revisited in 1994, when Reed and Johnson investigated the effect of sequence structure on successful sequence learning. They suggested that with many sequences used in the literature (e.g., A. Cohen et al., 1990; Nissen & Bullemer, 1987), participants might not actually be learning the sequence itself because ancillary differences (e.g., how frequently each position occurs in the sequence, how frequently back-and-forth movements occur, average number of targets before each position has been hit at least once, etc.) have not been adequately controlled. Therefore, effects attributed to sequence learning may be explained by learning simple frequency information rather than the sequence structure itself. Reed and Johnson experimentally demonstrated that when second order conditional (SOC) sequences (i.e., sequences in which the target position on a given trial is dependent on the target position of the previous two trails) were used in which frequency information was carefully controlled (one SOC sequence used to train participants on the sequence and a different SOC sequence in place of a block of random trials to test whether performance was better on the trained compared to the untrained sequence), participants demonstrated successful sequence learning despite the complexity of the sequence. Results pointed definitively to successful sequence learning because ancillary transitional differences were identical between the two sequences and therefore could not be explained by simple frequency information. This result led Reed and Johnson to suggest that SOC sequences are ideal for studying implicit sequence learning because whereas participants often become aware of the presence of some sequence types, the complexity of SOCs makes awareness far more unlikely. Today, it is common practice to use SOC sequences with the SRT task (e.g., Reed & Johnson, 1994; Schendan, Searl, Melrose, & Stern, 2003; Schumacher & Schwarb, 2009; Schwarb & Schumacher, 2010; Shanks & Johnstone, 1998; Shanks, Rowland, & Ranger, 2005). Though some studies are still published without this control (e.g., Frensch, Lin, & Buchner, 1998; Koch & Hoffmann, 2000; Schmidtke & Heuer, 1997; Verwey & Clegg, 2005).

### Measures of explicit knowledge

Although researchers can try to optimize their SRT design so as to reduce the potential for explicit contributions to learning, explicit learning may still occur. Therefore, many researchers use questionnaires to evaluate an individual participant’s level of conscious sequence know-ledge after learning is complete (for a review, see Shanks & Johnstone, 1998). Early studies (e.g., Curran & Keele, 1993; Frensch et al., 1998; Frensch, Wenke, & Rünger, 1999; Nissen & Bullemer, 1987) relied on explicitly questioning participants about their sequence knowledge. Specifically, participants were asked, for example, what they believed the goal of the experiment to be, and whether they noticed that the targets followed a repeating sequence of screen locations. It has been argued that given particular research goals, verbal report can be the most appropriate measure of explicit knowledge (Rünger & Frensch, 2010), other measures, however, are also used. For example, some researchers have asked participants to identify different chunks of the sequence using forced-choice recognition questionnaires (e.g., Frensch et al., 1998, 1999; Schumacher & Schwarb, 2009). Free-generation tasks in which participants are asked to recreate the sequence by ma-king a series of button-push responses have also been used to assess explicit awareness (e.g., Schwarb & Schumacher, 2010; Willingham, 1999; Willingham, Wells, Farrell, & Stemwedel, 2000). Furthermore, Destrebecqz and Cleeremans (2001)have applied the principles of Jacoby’s (1991) process dissociation procedure to assess implicit and explicit influences of sequence learning (for a review, see Curran, 2001). Destrebecqz and Cleeremans proposed assessing implicit and explicit sequence awareness using both an inclusion and exclusion version of the free-generation task. In the inclusion task, participants recreate the sequence that was repeated during the experiment. In the exclusion task, participants avoid reproducing the sequence that was repeated during the experiment. In the inclusion condition, participants with explicit knowledge of the sequence will likely be able to reproduce the sequence at least in part. However, implicit knowledge of the sequence might also contribute to generation performance. Thus, inclusion instructions cannot separate the influences of implicit and explicit know-ledge on free-generation performance. Under exclusion instructions, however, participants who reproduce the learned sequence despite being instructed not to are likely accessing implicit knowledge of the sequence. This clever adaption of the process dissociation procedure may provide a more accurate view of the contributions of implicit and explicit knowledge to SRT performance and is recommended. Despite its potential and relative ease to administer, this approach has not been used by many researchers.

## Measuring Sequence Learning

One last point to consider when designing an SRT experiment is how best to assess whether or not learning has occurred. In Nissen and Bullemer’s (1987) original experiments, between-group comparisons were used with some participants exposed to sequenced trials and others exposed only to random trials. A more common practice today, however, is to use a within-subject measure of sequence learning (e.g., A. Cohen et al., 1990; Keele, Jennings, Jones, Caulton, & Cohen, 1995; Schumacher & Schwarb, 2009; Willingham, Nissen, & Bullemer, 1989). This is accomplished by giving a participant several blocks of sequenced trials and then presenting them with a block of alternate-sequenced trials (alternate-sequenced trials are typically a different SOC sequence that has not been previously presented) before returning them to a final block of sequenced trials. If participants have acquired knowledge of the sequence, they will perform less quickly and/or less accurately on the block of alternate-sequenced trials (when they are not aided by knowledge of the underlying sequence) compared to the surrounding blocks of sequenced trials. This RT relationship, known as the *transfer effect*, is now the standard way to measure sequence learning in the SRT task.

With a foundational understanding of the basic structure of the SRT task and those methodological considerations that impact successful implicit sequence learning, we can now look at the sequence learning literature more carefully. It should be evident at this point that there are a number of task components (e.g., sequence structure, single- vs. dual-task learning environment) that influence the successful learning of a sequence. However, a primary question has yet to be addressed: What specifically is being learned during the SRT task? The next section considers this issue directly.

## Identifying the Locus of Sequence Learning

There are three main hypotheses^1^ in the SRT task literature concerning the locus of sequence learning: a stimulus-based hypothesis, a stimulus-response (S-R) rule hypothesis, and a response-based hypothesis. Each of these hypotheses maps roughly onto a different stage of cognitive processing (cf. Donders, 1969; Sternberg, 1969). Although cognitive processing stages are not often emphasized in the SRT task literature, this framework is typical in the broader human performance literature. This framework assumes at least three processing stages: When a stimulus is presented, the participant must encode the stimulus, select the task appropriate response, and finally must execute that response. Many researchers have proposed that these stimulus encoding, response selection, and response execution processes are organized as serial and discrete stages (e.g., Donders, 1969; Meyer & Kieras, 1997; Sternberg, 1969), but other organizations (e.g., parallel, serial, continuous, etc.) are possible (cf. Ashby, 1982; McClelland, 1979). It is possible that sequence learning can occur at one or more of these information-processing stages. We believe that consideration of information processing stages is critical to understanding sequence learning and the three main accounts for it in the SRT task.

The stimulus-based hypothesis states that a sequence is learned via the formation of stimulus-stimulus associations thus implicating the stimulus encoding stage of information processing. The stimulus-response rule hypothesis emphasizes the significance of linking perceptual and motor components thus implicating a central response selection stage (i.e., the cognitive process that activates representations for appropriate motor responses to particular stimuli, given one’s current task goals; Duncan, 1977; Kornblum, Hasbroucq, & Osman, 1990; Meyer & Kieras, 1997). And finally, the response-based learning hypothesis highlights the contribution of motor components of the task suggesting that response-response associations are learned thus implicating the response execution stage of information processing. Each of these hypotheses is briefly described below.

### Stimulus-based hypothesis

The stimulus-based hypothesis of sequence learning suggests that a sequence is learned via the formation of stimulus-stimulus associations and is not dependent on response (A. Cohen et al., 1990; Curran, 1997). More specifically, this hypothesis states that learning is stimulus-specific(Howard, Mutter, & Howard, 1992), effector-independent (A. Cohen et al., 1990; Keele et al., 1995; Verwey & Clegg, 2005), non-motoric (Grafton, Salidis, & Willingham, 2001; Mayr, 1996) and purely perceptual (Howard et al., 1992). Sequence learning will occur regardless of what type of response is made and even when no response is made at all (e.g., Howard et al., 1992; Mayr, 1996; Perlman & Tzelgov, 2009).

A. Cohen et al. (1990, Experiment 2) were the first to demonstrate that sequence learning is effector-independent. They trained participants in a dual-task version of the SRT task (simultaneous SRT and tone-counting tasks) requiring participants to respond using four fingers of their right hand. After 10 training blocks, they provided new instructions requiring participants to respond with their right index finger only. The amount of sequence learning did not change after switching effectors. The authors interpreted these data as evidence that sequence knowledge depends on the sequence of stimuli presented independently of the effector system involved when the sequence was learned (viz., finger vs. arm).

Howard et al. (1992) provided additional support for the non-motoric account of sequence learning. In their experiment participants either performed the standard SRT task (respond to the location of presented targets) or merely watched the targets appear without ma-king any response. After three blocks, all participants performed the standard SRT task for one block. Learning was tested by introducing an alternate-sequenced transfer block and both groups of participants showed a substantial and equivalent transfer effect. This study thus showed that participants can learn a sequence in the SRT task even when they do not make *any* response. However, Willingham (1999) has suggested that group differences in explicit knowledge of the sequence may explain these results; and thus these results do not isolate sequence learning in stimulus encoding. We will explore this issue in detail in the next section.

In another attempt to distinguish stimulus-based learning from response-based learning, Mayr (1996, Experiment 1) conducted an experiment in which objects (i.e., black squares, white squares, black circles, and white circles) appeared in four spatial locations. Both the object presentation order and the spatial presentation order were sequenced (different sequences for each). Participants always responded to the identity of the object. RTs were slower (indicating that learning had occurred) both when only the object sequence was randomized and when only the spatial sequence was randomized. These data support the perceptual nature of sequence learning by demonstrating that the spatial sequence was learned even when responses were made to an unrelated aspect of the experiment (object identity). However, Willingham and colleagues (Willingham, 1999; Willingham et al., 2000) have suggested that fixating the stimulus locations in this experiment required eye movements. Therefore, S-R rule associations may have developed between the stimuli and the ocular-motor responses required to saccade from one stimulus location to another and these associations may support sequence learning.

Although the data presented in this section are all consistent with a stimulus-based hypothesis of sequence learning, an alternative interpretation might be proposed. It is possible that stimulus repetition may leadto a processing short-cut that bypasses the response selection stage entirely thus speeding task performance (Clegg, 2005; cf. J. Miller, 1987; Mordkoff & Halterman, 2008). This idea is similar to the automatic-activation hypothesis prevalent in the human performance literature. This hypothesis states that with practice, the response selection stage can be bypassed and performance can be supported by direct associations between stimulus and response codes (e.g., Ruthruff, Johnston, & van Selst, 2001). According to Clegg, altering the pattern of stimulus presentation disables the shortcut resulting in slower RTs. In this view, learning is specific to the stimuli, but not dependent on the characteristics of the stimulus sequence (Clegg, 2005; Pashler & Baylis, 1991).

### Response-based hypothesis

Although there is support for the stimulus-based nature of sequence learning, there is also evidence for response-based sequence learning (e.g., Bischoff-Grethe, Geodert, Willingham, & Grafton, 2004; Koch & Hoffmann, 2000; Willingham, 1999; Willingham et al., 2000). The response-based hypothesis proposes that sequence learning has a motor component and that both making a response and the location of that response are important when learning a sequence.

As previously noted, Willingham (1999, Experiment 1) hypothesized that the results of the Howard et al. (1992) experiment were a product of the large number of participants who learned the sequence explicitly. It has been suggested that implicit and explicit learning are fundamentally different (N. J. Cohen & Eichenbaum, 1993; A. S. Reber et al., 1999) and are mediated by different cortical processing systems (Clegg et al., 1998; Keele et al., 2003; A. S. Reber et al., 1999). Given this distinction, Willingham replicated Howard and colleagues study and analyzed the data both including and excluding participants showing evidence of explicit knowledge. When these explicit learners were included, the results replicated the Howard et al. findings (viz., sequence learning when no response was required). However, when explicit learners were removed, only those participants who made responses throughout the experiment showed a significant transfer effect. Willingham concluded that when explicit knowledge of the sequence is low, knowledge of the sequence is contingent on the sequence of motor responses.

In an additional experiment, Willingham (1999; Experiment 3) provided further support for a response-based mechanism under-lying sequence learning. Using the SRT task, participants were trained, and showed significant sequence learning with a sequence requiring indirect manual responses in which they responded with the button one location to the right of the target (where if the target appeared in the right most location the left most finger was used to respond; training phase). After training was complete, participants switched to a direct S-R mapping in which they responded with the finger directly corresponding to the target position (testing phase). During the testing phase, either the sequence of responses (response constant group) or the sequence of stimuli (stimulus constant group) was maintained. Results indicated that the response constant group, but not the stimulus constant group, showed significant learning. Because maintaining the sequence structure of the stimuli from training phase to testing phase did not facilitate sequence learning but maintaining the sequence structure of the responses did, Willingham concluded that response processes (viz., learning of response locations) mediate sequence learning.

Thus, Willingham and colleagues (e.g., Willingham, 1999; Willingham et al., 2000) have provided considerable support for the idea that spatial sequence learning is based on the learning of the ordered response locations. It should be noted, however, that although other authors agree that sequence learning may depend on a motor component, they conclude that sequence learning is not restricted to the learning of the location of the response but rather the order of responses regardless of location (e.g., Goschke, 1998; Richard, Clegg, & Seger, 2009).

### Stimulus-response rule hypothesis

Finally, the S-R rule hypothesis of sequence learning offers yet another perspective on the possible locus of sequence learning. This hypo-thesis suggests that S-R rules and response selection are critical aspects of learning a sequence (e.g., Deroost & Soetens, 2006; Hazeltine, 2002; Schumacher & Schwarb, 2009; Schwarb & Schumacher, 2010; Willingham et al., 1989) emphasizing the significance of both perceptual and motor components. In this sense, the S-R rule hypothesis does for the SRT literature what the theory of event coding (Hommel, Musseler, Aschersleben, & Prinz, 2001) did for the perception-action literature linking perceptual information and action plans into a common representation. The S-R rule hypothesis asserts that sequence learning is mediated by the association of S-R rules in response selection. We believe that this S-R rule hypothesis provides a unifying framework for interpreting the seemingly inconsistent findings in the literature.

According to the S-R rule hypothesis of sequence learning, sequences are acquired as associative processes begin to link appropriate S-R pairs in working memory (Schumacher & Schwarb, 2009; Schwarb & Schumacher, 2010). It has previously been proposed that appropriate responses must be selected from a set of task-relevant S-R pairs active in working memory (Curtis & D’Esposito, 2003; E. K. Miller & J. D. Cohen, 2001; Pashler, 1994b; Rowe, Toni, Josephs, Frackowiak, & Passingham, 2000; Schumacher, Cole, & D’Esposito, 2007). The S-R rule hypothesis states that in the SRT task, selected S-R pairs remain in memory across several trials. This co-activation of multiple S-R pairs allows cross-temporal contingencies and associations to form between these pairs (N. J. Cohen & Eichenbaum, 1993; Frensch, Buchner, & Lin, 1994). However, while S-R associations are essential for sequence learning to occur, S-R rule sets also play an important role. In 1977, Duncan first noted that S-R mappings are governed by systems of S-R rules rather than by individual S-R pairs and that these rules are applicable to numerous S-R pairs. He further noted that with a rule or system of rules, “spatial transformations” can be applied. Spatial transformations hold some fixed spatial relation constant between a stimulus and given response. A spatial transformation can be applied to any stimulus and the associated response will bear a fixed relationship based on the original S-R pair. According to Duncan, this relationship is governed by a very simple relationship: *R = T(S)* where *R* is a given response, *S* is a given stimulus, and *T* is the fixed spatial relationship between them. For example, in the SRT task, if *T* is “respond one spatial location to the right,” participants can easily apply this transformation to the governing S-R rule set and do not need to learn new S-R pairs.

Shortly after the introduction of the SRT task, Willingham, Nissen, and Bullemer (1989; Experiment 3) demonstrated the importance of S-R rules for successful sequence learning. In this experiment, on each trial participants were presented with one of four colored *X*s at one of four locations. Participants were then asked to respond to the color of each target with a button push. For some participants, the colored *X*s appeared in a sequenced order, for others the series of locations was sequenced but the colors were random. Only the group in which the relevant stimulus dimension was sequenced (viz., the colored *X*s) showed evidence of learning. All participants were then switched to a standard SRT task (responding to the location of non-colored *X*s) in which the spatial sequence was maintained from the previous phase of the experiment. None of the groups showed evidence of learning. These data suggest that learning is neither stimulus-based nor response-based. Instead, sequence learning occurs in the S-R associations required by the task.

Soon after its introduction, the S-R rule hypothesis of sequence learning fell out of favor as the stimulus-based and response-based hypotheses gained popularity. Recently, however, researchers have developed a renewed interest in the S-R rule hypothesis as it seems to offer an alternative account for the discrepant data in the literature. Data has begun to accumulate in support of this hypothesis. Deroost and Soetens (2006), for example, demonstrated that when complicated S-R mappings (i.e., ambiguous or indirect mappings) are required in the SRT task, learning is enhanced. They suggest that more complex mappings require more controlled response selection processes, which facilitate learning of the sequence. Unfortunately, the specific mechanism underlying the importance of controlled processing to robust sequence learning is not discussed in the paper. The importance of response selection in successful sequence learning has also been demonstrated using functional magnetic resonance imaging (fMRI; Schwarb & Schumacher, 2009). In this study we orthogonally manipulated both sequence structure (i.e., random vs. sequenced trials) and response selection difficulty (i.e., direct vs. indirect mapping) in the SRT task. These manipulations independently activated largely overlapping neural systems indicating that sequence and S-R compatibility may rely on the same fundamental neurocognitive processes (viz., response selection).

Furthermore, we have recently demonstrated that sequence learningpersists across an experiment even when the S-R mapping is altered, so long as the same S-R rules or a simple transformation of the S-R rules (e.g., shift response one position to the right) can be applied (Schwarb & Schumacher, 2010). In this experiment we replicated the findings of the Willingham (1999, Experiment 3) study (described above) and hypothesized that in the original experiment, when the response sequence was maintained throughout, learning occurred because the mapping manipulation did not significantly alter the S-R rules required to perform the task. We then repeated the experiment using a substantially more complex indirect mapping that required entirely different S-R rules from those required of the direct mapping. Learning was disrupted when the S-R mapping was altered even when the sequence of stimuli or the sequence of responses was maintained. Together these results indicate that only when the same S-R rules were applicable across the course of the experiment did learning persist.

### An S-R rule reinterpretation

Up to this point we have alluded that the S-R rule hypothesis can be used to reinterpret and integrate inconsistent findings in the literature. We expand this position here and demonstrate how the S-R rule hypothesis can explain many of the discrepant findings in the SRT literature.

Studies in support of the stimulus-based hypothesis that demonstrate the effector-independence of sequence learning (A. Cohen et al., 1990; Keele et al., 1995; Verwey & Clegg, 2005) can easily be explained by the S-R rule hypothesis. When, for example, a sequence is learned with three-finger responses, a set of S-R rules is learned. Then, if participants are asked to begin responding with, for example, one finger (A. Cohen et al., 1990), the S-R rules are unaltered. The same response is made to the same stimuli; just the mode of response is different, thus the S-R rule hypothesis predicts, and the data support, successful learning. This conceptualization of S-R rules explains successful learning in a number of existing studies. Alterations like changing effector (A. Cohen et al., 1990; Keele et al., 1995), switching hands (Verwey & Clegg, 2005), shifting responses one position to the left or right (Bischoff-Grethe et al., 2004; Willingham, 1999), changing response modalities (Keele et al., 1995), or using a mirror image of the learned S-R mapping (Deroost & Soetens, 2006; Grafton et al., 2001) do not require a new set of S-R rules, but merely a transformation of the previously learned rules. When there is a transformation of one set of S-R associations to another, the S-R rules hypothesis predicts sequence learning.

The S-R rule hypothesis can also explain the results obtained by advocates of the response-based hypothesis of sequence learning. Willingham (1999, Experiment 1) reported when participants only watched sequenced stimuli presented, learning did not occur. However, when participants were required to respond to those stimuli, the sequence was learned. According to the S-R rule hypothesis, participants who only observe a sequence do not learn that sequence because S-R rules are not formed during observation (provided that the experimental design does not permit eye movements). S-R rules can be learned, however, when responses are made. Similarly, Willingham et al. (2000, Experiment 1) conducted an SRT experiment in which participants responded to stimuli arranged in a lopsided diamond pattern using one of two keyboards, one in which the buttons were arranged in a diamond and the other in which they were arranged in a straight line. Participants used the index finger of their dominant hand to make all responses. Willingham and colleagues reported that participants who learned a sequence using one keyboard and then switched to the other keyboard show no evidence of having previously learned the sequence. The S-R rule hypothesis says that there are no correspondences between the S-R rules required to perform the task with the straight-line keyboard and the S-R rules required to perform the task with the diamond keyboard. The tasks are too dissimilar and therefore a mere spatial transformation of the S-R rules originally learned is not sufficient to transfer sequence knowledge acquired during training.

Thus, although there are three prominent hypotheses concerning the locus of sequence learning and data supporting each, the literature may not be as incoherent as it initially seems. Recent support for the S-R rule hypothesis of sequence learning provides a unifying framework for reinterpreting the various findings in support of other hypotheses. It should be noted, however, that there are some data reported in the sequence learning literature that cannot be explained by the S-R rule hypothesis. For example, it has been demonstrated that participants can learn a sequence of stimuli and a sequence of responses simultaneously (Goschke, 1998) and that simply adding pauses of varying lengths between stimulus presentations can abolish sequence learning(Stadler, 1995). Thus further research is required to explore the strengths and limitations of this hypothesis. Still, the S-R rule hypo-thesis provides a cohesive framework for much of the SRT literature. Furthermore, implications of this hypothesis on the importance of response selection in sequence learning are supported in the dual-task sequence learning literature as well.

## Dual-Task Sequence Learning

Even in the first SRT study, the effect of dividing attention (by performing a secondary task) on sequence learning was investigated (Nissen & Bullemer, 1987). Since then, there has been an abundance of research on dual-task sequence learning, however, the results of this effort have been controversial with many studies reporting intact sequence learning under dual-task conditions (e.g., Frensch et al., 1998; Frensch & Miner, 1994; Grafton, Hazeltine, & Ivry, 1995; Jiménez & Vázquez, 2005; Keele et al., 1995; McDowall, Lustig, & Parkin, 1995; Schvaneveldt & Gomez, 1998; Shanks & Channon, 2002; Stadler, 1995) and others reporting impaired learning with a secondary task (e.g., Heuer & Schmidtke, 1996; Nissen & Bullemer, 1987). As a result, several hypotheses have emerged in an attempt to explain these data and provide general principles for understanding multi-task sequence learning. These hypotheses include the attentional resource hypothesis (Curran & Keele, 1993; Nissen & Bullemer, 1987), the automatic learning hypothesis/suppression hypothesis (Frensch, 1998; Frensch et al., 1998, 1999; Frensch & Miner, 1994), the organizational hypothesis (Stadler, 1995), the task integration hypothesis (Schmidtke & Heuer, 1997), the two-system hypothesis (Keele et al., 2003), and the parallel response selection hypothesis (Schumacher & Schwarb, 2009) of sequence learning. While these accounts seek to characterize dual-task sequence learning rather than identify the underlying locus of this learning, connections can still be drawn. We propose that the parallel response selection hypothesis is not only consistent with the S-R rule hypothesis of sequence learning discussed above, but also most ade-quately explains the existing literature on dual-task spatial sequence learning.

### Methodology for studying dual-task sequence learning

Before examining these hypotheses, however, it is important to understand the specifics of the method used to study dual-task sequence learning. The secondary task typically used by researchers when studying multi-task sequence learning in the SRT task is a tone-counting task. In this task, participants hear one of two tones on each trial. They must keep a running count of, for example, the high tones and must report this count at the end of each block. This task is frequently used in the literature because of its efficacy in disrupting sequence learning while other secondary tasks (e.g., verbal and spatial working memory tasks) are ineffective in disrupting learning (e.g., Heuer & Schmidtke, 1996; Stadler, 1995). The tone-counting task, however, has been criticized for its complexity (Heuer & Schmidtke, 1996). In this task participants must not only discriminate between high and low tones, but also continuously update their count of those tones in working memory. Therefore, this task requires many cognitive processes (e.g., selection, discrimination, updating, etc.) and some of these processes may interfere with sequence learning while others may not. Additionally, the continuous nature of the task makes it difficult to isolate the various processes involved because a response is not required on each trial (Pashler, 1994a). However, despite these disadvantages, the tone-counting task is frequently used in the literature and has played a prominent role in the development of the various theirs of dual-task sequence learning.

### Accounts of dual-task sequence learning

The attentional resource hypothesis of dual-task sequence learning stems from early work using the SRT task (e.g., Curran & Keele, 1993; Nissen & Bullemer, 1987) and proposes that implicit learning is eliminated under dual-task conditions due to a lack of attention available to support dual-task performance and learning concurrently. In this theory, the secondary task diverts attention from the primary SRT task and because attention is a finite resource (cf. Kahneman, 1973), learning fails. Later A. Cohen et al. (1990) refined this theory noting that dual-task sequence learning is impaired only when sequences have no unique pairwise associations (e.g., ambiguous or second order conditional sequences). Such sequences require attention to learn because they cannot be defined based on simple associations.

In stark opposition to the attentional resource hypothesis is the automatic learning hypothesis (Frensch & Miner, 1994) that states that learning is an automatic process that does not require attention. Therefore, adding a secondary task should not impair sequence learning. According to this hypothesis, when transfer effects are absent under dual-task conditions, it is not the learning of the sequence that is impaired, but rather the expression of the acquired knowledge is blocked by the secondary task (later termed the *suppression hypothesis*; Frensch, 1998; Frensch et al., 1998, 1999; Seidler et al., 2005). Frensch et al. (Frensch et al., 1998, Experiment 2a) provided clear support for this hypothesis. They trained participants in the SRT task using an ambiguous sequence under both single-task and dual-task conditions (secondary tone-counting task). After five sequenced blocks of trials, a transfer block was introduced. Only those participants who trained under single-task conditions demonstrated significant learning. However, when those participants trained under dual-task conditions were then tested under single-task conditions, significant transfer effects were evident. These data suggest that learning was successful for these participants even in the presence of a secondary task, however, it was only after the secondary task was removed that this learned knowledge was expressed.

Stadler (1995) noted that when a tone-counting secondary task is paired with the SRT task, updating is only required on a subset of trials (e.g., only when a high tone occurs). He suggested this variability in task requirements from trial to trial disrupted the organization of the sequence and proposed that this variability is responsible for disrupting sequence learning. This is the premise of the organizational hypothesis. He tested this hypothesis in a single-task version of the SRT task in which he inserted long or short pauses between presentations of the sequenced targets. He demonstrated that disrupting the organization of the sequence with pauses was sufficient to produce deleterious effects on learning similar to the effects of performing a simultaneous tone-counting task. He concluded that consistent organization of stimuli is critical for successful learning.

The task integration hypothesis states that sequence learning is frequently impaired under dual-task conditions because the human information processing system attempts to integrate the visual and auditory stimuli into one sequence (Schmidtke & Heuer, 1997). Because in the standard dual-SRT task experiment, tones are randomly presented, the visual and auditory stimuli cannot be integrated into a repetitive sequence. In their Experiment 1, Schmidtke and Heuer asked participants to perform the SRT task and an auditory go/no-go task simultaneously. The sequence of visual stimuli was always six positions long. For some participants the sequence of auditory stimuli was also six positions long (six-position group), for others the auditory sequence was only five positions long (five-position group) and for others the auditory stimuli were presented randomly (random group). For both the visual and auditory sequences, participant in the random group showed significantly less learning (i.e., smaller transfer effects) than participants in the five-position, and participants in the five-position group showed significantly less learning than participants in the six-position group. These data indicate that when integrating the visual and auditory task stimuli resulted in a long complicated sequence, learning was significantly impaired. However, when task integration resulted in a short less-complicated sequence, learning was successful.

Schmidtke and Heuer’s (1997) task integration hypothesis proposes a similar learning mechanism as the two-system hypothesis of sequence learning (Keele et al., 2003). The two-system hypothesis proposes a unidimensional system responsible for integrating information within a modality and a multidimensional system responsible for cross-modality integration. Under single-task conditions, both systems work in parallel and learning is successful. Under dual-task conditions, however, the multidimensional system attempts to integrate information from both modalities and because in the typical dual-SRT task the auditory stimuli are not sequenced, this integration attempt fails and learning is disrupted.

The final account of dual-task sequence learning discussed here is the parallel response selection hypothesis (Schumacher & Schwarb, 2009). It states that dual-task sequence learning is only disrupted when response selection processes for each task proceed in parallel. Schumacher and Schwarb conducted a series of dual-SRT task studies using a secondary tone-identification task, which is similar to the tone-counting task except that participants respond to each tone by saying “high” or “low” on every trial. Because participants respond to both tasks on each trail, researchers can investigate task processing organization (i.e., whether processing stages for the two tasks are performed serially or simultaneously). We demonstrated that when visual and auditory stimuli were presented simultaneously and participants attempted to select their responses simultaneously, learning did not occur. However, when visual and auditory stimuli were presented 750 ms apart, thus minimizing the amount of response selection overlap, learning was unimpaired (Schumacher & Schwarb, 2009, Experiment 1). These data suggested that when central processes for the two tasks are organized serially, learning can occur even under multi-task conditions. We replicated these findings by altering central processing overlap in different ways. In Experiment 2, visual and auditory stimuli were presented simultaneously, however, participants were either instructed to give equal priority to the two tasks (i.e., promoting parallel processing) or to give the visual task priority (i.e., promoting serial processing). Again sequence learning was unimpaired only when central processes were organized sequentially. In Experiment 3, the psychological refractory period procedure was used so as to introduce a response-selection bottleneck necessitating serial central processing. Data indicated that under serial response selection conditions, sequence learning emerged even when the sequence occurred in the secondary rather than primary task.

We believe that the parallel response selection hypothesis provides an alternate explanation for much of the data supporting the various other hypotheses of dual-task sequence learning. The data from Schumacher and Schwarb (2009) are not easily explained by any of the other hypotheses of dual-task sequence learning. These data provide evidence of successful sequence learning even when attention must be shared between two tasks (and even when they are focused on a nonsequenced task; i.e., inconsistent with the attentional resource hypothesis) and that learning can be expressed even in the presence of a secondary task (i.e., inconsistent with the suppression hypothesis).Additionally, these data provide examples of impaired sequence learning even when consistent task processing was required on each trial (i.e., inconsistent with the organizational hypothesis) and when only the SRT task stimuli were sequenced while the auditory stimuli were randomly ordered (i.e., inconsistent with both the task integration hypothesis and two-system hypothesis).

**Figure 1.**
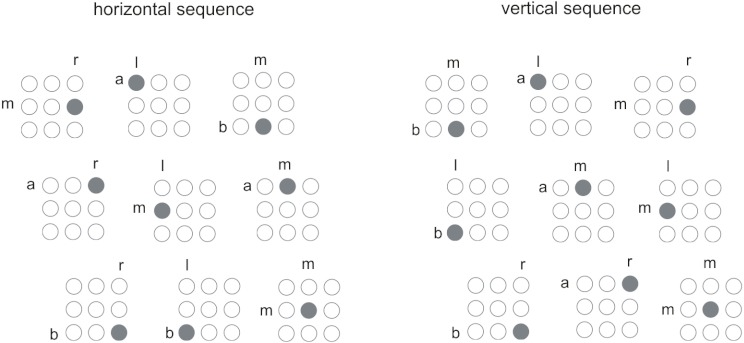
Analysis of dual-task interference on the serial reaction time (SRTSRT) task of 21 published dual-task sequence learning experiments. In each experiment, the SRT task was paired with a tone-counting task. For the SRT task, the underlying sequence was higher order (i.e., at least some ambiguous associations) and deterministic (i.e., no studies using probabilistic mappings were included). The dual-task interference on SRT task performance (i.e., the difference between the SRTSRT task reaction times [RTs] under single- and dual-task conditions) is indicated by the length of the white and black bars for each experiment. The numbers across the top of the figure represent ranges of approximate mean RTs. The left edge of each bar represents the approximate mean RTs for the single-task conditions. The right edge represents the approximate mean RTs for the dual-task conditions. Experiments reporting significant dual-task sequence learning are plotted with white bars and experiments reporting no significant dual-task sequence learning are plotted with black bars. The mean transfer effect (i.e., the amount of sequence learning) for each experiment is also shown. Adapted from “Parallel Response Selection Disrupts Sequence Learning Under Dual-Task Conditions” by E. H. Schumacher and H. Schwarb, 2009, *Journal of Experimental Psychology: General*, 138, p. 282. Copyright 2009 by the American Psychological Association. Reprinted with permission.

Furthermore, in a meta-analysis of the dual-task SRT literature (cf. Schumacher & Schwarb, 2009), we looked at average RTs on single-task compared to dual-task trials for 21 published studies investigating dual-task sequence learning (cf. Figure 1). Fifteen of those experiments reported successful dual-task sequence learning while six reported impaired dual-task learning. We examined the amount of dual-task interference on the SRT task (i.e., the mean RT difference between single- and dual-task trials) present in each experiment. We found that experiments that showed little dual-task interference were more likely to report intact dual-task sequence learning. Similarly, those studies showing large dual-task interference effects were more likely to report impaired dual-task sequence learning. In fact, there was significantly less dual-task interference in those studies demonstrating successful sequence learning compared to those studies demonstrating impaired learning. This meta-analysis suggests that high dual-task costs are associated with impaired sequence learning and that high dual-task costs are likely the result of parallel response selection processes in the dual-SRT task. However, when response selection processes occur serially and dual-task interference is minimized, sequence learning emerges. This hypothesis is consistent with the S-R rule hypothesis of sequence learning derived from the single-task SRT literature.

## Beyond the SRT Task

This review of the vast literature surrounding the SRT task demonstrates that the past 20 years of research have afforded great insights into the underlying structure of implicit sequence learning. However, the generalizability of these principles to other implicit learning tasks has yet to be determined. The SRT task provides a highly controlled and efficient procedure for modeling sequence learning behavior; however, the fidelity of the underlying processes to those of real-world sequential learning has yet to be verified (Mathews, 1997). Applying the knowledge acquired about implicit sequence learning from the SRT task to other related implicit learning task is an important first step in verifying the universality of these SRT-derived accounts for implicit sequence learning.

We have proposed here that the response selection stage is critical to successful sequence learning and that sequence learning is mediated by the association of S-R rules. We have demonstrated that this account can explain much of the data in the SRT literature; however, the question remains as to whether this account is also supported by implicit learning data from other tasks.

In addition to the SRT task, numerous other tasks have been used to investigate implicit learning. Some of these tasks are very similar to the SRT task, such as the triplet-learning task (e.g., Howard, Howard, Dennis, & Kelly, 2008) and the target-marked locations task (e.g., Remillard, 2003, 2009). Other tasks are less similar, such as artificial grammar learning (AGL) tasks (e.g., A. S. Reber, 1967; A. S. Reber & Allen, 1978; A. S. Reber et al., 1999), mirror tracing tasks (e.g., Grafton et al., 1995), serial search tasks (e.g., Goschke, 1998), prototype extraction tasks (e.g., Knowlton & Squire, 1993; Reed, Squire, Patalano, Smith, & Jonides, 1999), speeded choice tasks (e.g., Pashler & Baylis, 1991), weather prediction tasks (e.g., Knowlton, Squire, & Gluck, 1994), and dynamic system control tasks (e.g., Berry & Broadbent, 1984) to name a few. Among these various tasks, there is some evidence that the S-R rule hypothesis may generalize to other instances of implicit learning. However, for other tasks, the possible importance of S-R rules to successful performance has either not been supported or has yet to be evaluated.

One example of a task where the principles of the S-R rule hypothesis are applicable is the AGL task. Like the SRT task, the AGL task has been used frequently to study implicit learning (for reviews, see Cleeremans et al., 1998; Dienes & Berry, 1997). In the AGL task participants are asked to memorize a set of letter strings that have been constructed according to an artificial grammar (i.e., a finite-state language used to build strings of symbols, letters, numbers, shapes, etc., with consistent relations; for review, see Pothos, 2007). After learning is complete, participants are presented with new letter strings and asked to categorize them as either grammatical or ungrammatical. The standard finding is that the frequency with which participants classify grammatical strings as being a part of the learned grammar is significantly greater than chance (e.g., A. S. Reber, 1967). As in the SRT literature, there have been multiple theories developed in an attempt to explain AGL task data (for review, see Pothos, 2007). One hypothesis in particular shows marked similarity to the S-R rule hypothesis of sequence learning described previously; namely the rules hypothesis of artificial grammar learning (cf. Pothos, 2007). This hypothesis states that in the AGL task, participants learn the underlying rules that govern the memorized grammatical letter strings (A. S. Reber & Allen, 1978) and participants are then able to use knowledge of these rules to classify new letter strings as grammatical or not (e.g., A. S. Reber, 1967). When these abstract rule structures have been learned, participants can apply those rules to accurately classify not only new letter strings but also new letter sets (e.g., A. S. Reber, 1967). Thus, as predicted by the S-R rule hypothesis, the rules hypothesis suggests that one set of rules can be effectively applied to multiple stimuli. It is unlikely, however, that proponents of the rules hypothesis have conceptualized these overarching rules as S-R rules as in the artificial grammar paradigm, multiple stimuli require a single response; therefore, the nature of the rules in each account may not correspond directly.

Further support for the S-R rule hypothesis outside of the SRT literature comes from studies by Pashler and Baylis (1991) who in a series of experiments emphasized the importance of S-R rules in successful performance of a speeded choice task. In their experiment, digits, letters, and symbols were mapped onto three buttons from right to left (training phase). After several training trials with this mapping, participants were presented with other digits, letters, and symbols that were not presented during the training phase (testing phase). Despite the differences in stimuli, performance was not disrupted (Experiment 1)because the same rules (e.g., “if digit then rightmost button”) were applicable. Similarly, if during the testing phase participants were asked to respond to digits, letters, and symbols from left to right but with the opposite hand, learning was again undisrupted (Experiment 5) because the same rules still applied. However, if during the testing phase, digits, letters, and symbols were remapped to different fingers (middle, left, right buttons, respectively), performance was substantially impaired (Experiment 4) because the S-R rules were changed (e.g., “if digits then rightmost button” no longer produced the correct response). These data demonstrate that only when the S-R rules were altered from training to test was performance impaired in the speeded choice task.

Theories explaining the results of the weather prediction task (e.g., Knowlton et al., 1994) sometimes also show similarity to the S-R rule hypothesis. The weather prediction task is a probabilistic classification task (cf. Ashby & Maddox, 2005) in which on each trial participants are presented with one, two, or three cards marked with unique geometric patterns (four cards in all). The participants are asked to state whether or not the presented combination of cards indicates rain or sun and each combination is probabilistically associated with each outcome. There are multiple strategies that can be effectively used in this task (Ashby & Maddox, 2005):

1. Participants can respond based on the presence (or absence) of one particular card, thus relying on a single S-R rule to respond.

2. Participants can respond based on multiple cues thus requiring information integration processes.

3. When one card is presented, participants can learn what that card predicts (single S-R rule), respond accordingly, and then simply guess when multiple cards are presented (singleton strategy).

Gluck, Shohamy, and Myers (2002) investigated individual differences in strategy use in the weather prediction task and determined that the vast majority of participants (about 80-90% in their studies) used the singleton strategy in the early phases of the experiment and only shifting toward a multiple cue strategy later in training. Gluck and colleagues believed that only the multiple-cue strategy involves rule-based learning, therefore they concluded that although rule-based learning can occur in the weather prediction task, it is not the most commonly adopted strategy. However, as we have suggested, the singleton strategy could also be interpreted as a rule-based approach, though an inefficient and impoverished one. If using the singleton strategy participants are learning a single S-R association for a single card, when that card is presented they can always apply that rule. On multiple card trials, this strategy may not result in an error response, however, it is still consistent with rule-use. Thus, the S-R rule hypothesis may be more relevant in the weather prediction task than originally believed.

Despite support for the S-R rule hypothesis in several implicit learning tasks, other tasks demonstrate that S-R rules may not be critically important to learning in every case. For example, in the dynamic system control task, participants engage in a computer simulation (e.g., a sugar factory simulation; Berry & Broadbent, 1984) where participants attempt to control some output (e.g., total sugar production) by manipulating various input variables (e.g., the number of workers). With practice, performance improves indicating that participants have learned to control the system. Dienes and Fahey (1995), however, demonstrated that participants performed well when situations were repeated and they could simply replicate the response that had been successful previously. When presented with new situations, however, participants performed at chance levels. These data indicate that learning and successful performance in this task is associated with particular items rather than with underlying rules (Dienes & Berry, 1997; Dienes & Fahey, 1995).

Another instance where the S-R rule hypothesis is insufficient can be seen in a study by Goschke (1998) who demonstrated that performance on a serial search task could not be explained by learning the underlying S-R rules. In this study, participants were presented with four letters and an auditory cue on each trial. The auditory cue indicated the letter to which participants were to respond. Both the auditory stimuli and the required responses composed different sequences. Participants were able to learn both sequences simultaneously. The S-R rule hypothesis did not predict learning of the auditory sequence in this experiment. The auditory stimulus cued which letter stimulus to focus on and the letter stimulus dictated the appropriate response. Thus the auditory S-R pairings changed on each trial and no general rules governed this relationship; therefore, the S-R rule hypothesis predicts that learning of the auditory sequence should not occur. However it should be noted that these data are inconsistent with other reports in which participants failed to learn two sequences simultaneously (e.g., Mayr, 1996; Schmidtke & Heuer, 1997).

Additionally, research has shown that performance on the prototype extraction task does not appear to be governed by S-R rule-based learning (for review, see Ashby & Maddox, 2005). In this task, participants are presented with, for example, a series of dot patterns (training phase). These patterns are created by distorting a prototype image (e.g., nine dots randomly distributed in a 12 × 12 cm area) to varying degrees (low- and high-level distortions); however, the prototype is not presented during training. After the training phase is complete, participants are presented with more nine-dot patterns (some previously seen and some new, including the prototype) and asked to determine whether or not the pattern belongs to the category of stimuli seen duringthe training phase. Typically participants endorse the unstudied prototype with the highest probability followed by low-level distortions and then high-level distortions and random patterns (e.g., Knowlton & Squire, 1993). Results from this task are typically explained with exemplar and prototype theories and are contrasted with rule-based category learning (for review, see Ashby & Maddox, 2005). Neuroimaging data demonstrating differential activity in the visual cortex (i.e., bilateral posterior occipital cortex) to categorical versus noncategorical stimuli have provided an alternate account suggesting that perceptual learning likely plays an important role in successful performance on these prototype extraction tasks (Ashby & Maddox, 2005; P. J. Reber, Stark, & Squire, 1998). These data thus suggest category learning occurs prior to the response selection stage in the prototype extraction ask.

Thus it is evident that there is some support for S-R rule based learning in the SRT task and many other implicit learning tasks. However, there are other tasks widely used to investigate the underlying neurocognitive mechanisms involved in implicit learning that do not rely on S-R rules. Although there is some indication that S-R rule learning can explain performance on a variety of implicit learning tasks, further research is necessary to truly assess the generalizability of this hypothesis. Such future research constitutes an important step in trying to identify a unifying theory of implicit learning that is more generally applicable and broad in scope rather than highly task specific.

## Conclusions

In this review we have presented the SRT task in detail with a particular focus on important factors to consider when designing an SRT study. We have summarized the various hypotheses associated with identifying the locus of spatial sequence learning and have demonstrated how the S-R rule hypothesis provides a cohesive framework for unifying a seemingly incongruous literature. Additionally we have reviewed various studies using the dual-SRT task and suggested that the parallel response selection hypothesis can explain many of the discrepant findings in this literature. The S-R rule hypothesis and the parallel response selection hypothesis are conceptually similar and both highlight the importance of response selection processes in successful sequence learning. We propose that taken together, the S-R rule hypothesis and parallel response selection hypothesis not only provide a unifying framework, but also point to response selection as the underlying critical cognitive process for effective sequence learning.

Finally, much has been learned about the underlying cognitive processes that support implicit spatial sequence learning in the SRT task, however, the generalizability of the knowledge and understanding gleaned with this paradigm has often been ignored. A wide variety of tasks have been used to study implicit learning and there is a need in the literature to attempt to identify congruences across these tasks that will likely tell us about implicit learning more generally. We hope, therefore, that this review serves as a challenge to researchers to widen our perspectives and apply what we have learned from the SRT task to other implicit learning domains in an attempt to understand implicit learning more broadly.

## Footnotes

1 This is not an exhaustive list of accounts; however, these are the most frequently discussed hypotheses regarding the locus of sequence learning in the SRT task. There is an additional plausible account that might be important for sequence learning (viz., the response-effect account), however, the nature of the SRT task makes it impossible to disambiguate the response-effect account from the S-R rule account. The response-effect account of sequence learning (e.g., Ziessler, 1998; Ziessler & Nattkemper, 2001) states that response-stimulus (R-S) learning is a powerful and the major mechanism in successful sequence learning. This theory states that when participants are presented with a spatial sequence, learning and performance improvements depend on learning the relationship between a current response and the following target location (Ziessler, 1998; Ziessler & Nattkemper, 2001). Much data in support of this theory come from the serial search-and-reaction task in which various letters are presented in a 5 × 5 matrix. Letter stimuli and button push responses are organized in an eight-to-four S-R mapping so that the relationship between response and upcoming target location can be systematically varied. Data demonstrate a performance improvement when a response predicts the following target location even when the presentation order of stimuli and required responses are random (Ziessler, 1998). Unfortunately, the data from the SRT task are unable to address this theory directly because with the one-to-one stimulus response mapping used here, R-S order is confounded with both the stimulus and response sequence.
